# RIG-I Like Receptors in Antiviral Immunity and Therapeutic Applications

**DOI:** 10.3390/v3060906

**Published:** 2011-06-23

**Authors:** Reneé C. Ireton, Michael Gale

**Affiliations:** Department of Immunology, University of Washington School of Medicine, Seattle, WA 98195, USA; E-Mail: rireton@u.washington.edu

**Keywords:** RIG-I, inflammation, adjuvant

## Abstract

The RNA helicase family of RIG-I-like receptors (RLRs) is a key component of host defense mechanisms responsible for detecting viruses and triggering innate immune signaling cascades to control viral replication and dissemination. As cytoplasm-based sensors, RLRs recognize foreign RNA in the cell and activate a cascade of antiviral responses including the induction of type I interferons, inflammasome activation, and expression of proinflammatory cytokines and chemokines. This review provides a brief overview of RLR function, ligand interactions, and downstream signaling events with an expanded discussion on the therapeutic potential of targeting RLRs for immune stimulation and treatment of virus infection.

## 1. Introduction

Cell-intrinsic innate immune defenses are our first line of resistance against infections caused by harmful pathogens. Both immune and non-immune cells alike are armed with the ability to detect pathogens and alert the innate immune system of their presence. In recent years, pattern recognition receptors (PRRs), such as Toll-like receptors (TLRs), RIG-I-like receptors (RLRs), and Nod-like receptors (NLRs), have been defined as factors that recognize pathogen-associated molecular patterns (PAMPs). PAMPs are non-self motifs embedded in the molecular structure of pathogen-specific macromolecules and are recognized as non-self through interaction with certain PRRs. PAMP-PRR interactions lead to induction of signaling events that activate innate immunity [1]. The various PRRs are differentially expressed within the cellular compartments and demonstrate specific affinities for various pathogen-intrinsic molecular structures. In particular, the RLR family of PRRs has emerged as a key cellular sensor of RNA virus infection. Once activated by binding to an RNA PAMP ligand, the RLRs trigger a multitude of downstream signaling cascades that lead to antiviral host responses, such as the induction of type-I interferons, inflammasome activation, and the expression of proinflammatory cytokines and chemokines. This review focuses specifically on RLR mechanism of action, potential methods of cellular surveillance, and the role of RLR agonists in possible new therapeutic applications.

## 2. Structure and Functional Mechanisms of RLRs

The RLR family of PRRs is comprised of three members: Retinoic acid-inducible gene I (RIG-I), melanoma differentiation-associated gene 5 (MDA5), and laboratory of genetics and physiology 2 (LGP2). All members contain a centrally located DExH-box helicase domain, while only RIG-I and MDA5, but not LGP2, have two caspase recruitment domains (CARDs) located at the N-terminus. In contrast, both RIG-I and LGP2 contain a C-terminal repressor domain that regulates signaling by holding the molecule in a closed, signaling-off state until ligand binding leads to its altered conformation of a signaling-on state that drives antiviral defenses [2].

The RLRs reside in the cytoplasm, where they survey cellular contents for the presence of RNA viruses [2]. As detailed descriptions of the signaling cascades activated by RLRs have been provided in previous reviews [3–5], we provide only a brief description of the RIG-I signaling process below. In the presence of RNA ligand binding, an ATP-dependent conformational change occurs and the activated CARD domains are released from signaling repression to induce signaling by interacting with the adaptor molecule, IPS-1 (also known as MAVS, VISA, and Cardif). Activation of IPS-1, in turn triggers signaling cascades involving TRAF3/6, caspase 8/10, RIP-1, Fas-associated death domain (FADD), and TNF receptor-associated death domain (TRADD). These signaling cascades ultimately activate the kinase activity of the TBK1/IKK-ɛ and IKKα/IKKβ complexes to induce transcription of type-I IFNs and proinflammatory cytokines by activating the NF-κB and IRF-3 transcription factors [4]. A large number of recent studies have begun to identify the varied mechanisms by which the RLR signaling pathway is modulated and have identified a variety of RLR interacting/signaling partners. These factors include adaptor and signaling proteins such as TRAF3, TRAF2, TRAF6, TANK, SIKE, NEMO/IKKγ, FADD, RIP1, TRADD, caspase 8/10, and STING/MITA/MPYS as well as negative signaling regulators such as RNF125, ISG15, DAK, Atg12-Atg5, NLRX1, caspase-1, and several deubiquitinating enzymes (reviewed in detail in [6,7]). Indeed, other studies have shown that RIG-I is directly mediated through ubiquitination or the interaction with free ubiquitin [8,9].

Together with the RLRs, these factors serve to direct downstream signaling that drives IFN expression and secretion from the virus-infected cell. Secreted IFN drives both autocrine and paracrine immune signaling in the infected cell and surrounding cells in the local tissue, respectively. Activation of the interferon receptor stimulates the JAK-STAT pathway, which ultimately induces the nuclear localization of ISGF3, the central transcription factor responsible for promoting high expression of interferon stimulated genes (ISGs). This second wave of transcriptional activity directed by IFN to initiate ISG expression creates a powerful immune response. The hundreds of ISGs encoded by the human genome [10] have yet to be thoroughly examined and functionally characterized, but the protein products of a subset of these genes are known to be potent immunomodulators and antivirals. Viewed in this light, pathways such as the RLRs that lead to the triggering of ISG induction become interesting targets for designing therapeutics aimed at inducing the powerful ISG response.

## 3. Ligand Recognition by RLRs

RIG-I and MDA5 are essential and sufficient for the induction of innate immune defenses and type-I interferon responses to RNA viruses, as cells lacking both factors failed to produce type-I IFNs when exposed to certain RNA viruses [11,12]. However, with the exception of reoviruses, paramyxoviruses, and flaviviruses including Dengue virus and West Nile virus; RIG-I and MDA5 each trigger innate antiviral response and IFN expression in response to specific sets of viruses. The virus specificity of RIG-I and MDA5 is intrinsic to the RNA species they detect: RIG-I detects RNA marked with 5′ triphosphates (5′ppp) and containing short dsRNA structure motifs and/or poly-uridine motifs that mark an RNA as non-self [13–18]. MDA5 is thought to bind to activated long, stable dsRNA structures such as RNA replication intermediates that might be hybridized to genome RNA during infection [11]. RIG-I detects both positive and negative strand RNA viruses including Japanese encephalitis virus, Sendai virus, vesicular stomatitis virus, influenza virus, Hepatitis C virus, respiratory syncytial virus, and Newcastle disease virus; while MDA5 has been shown to specifically detect picornaviruses such as encephalomyocarditis virus, Mengo virus, and Theiler’s virus. More recent work has found that RIG-I and MDA5 can also detect rotavirus in infected intestinal epithelium [19,20]. Importantly, MDA5 also appears to be the primary RLR for detecting poly(I:C), the memetic of dsRNA. RIG-I also recognizes cellular RNA polymerase III-derived RNA that can be generated from viral DNA templates, thereby linking RIG-I signaling with DNA virus infection [21]. Table 1 features the ligands known to be recognized by each RLR family member.

The ligand recognition properties of LGP2 are less well-defined, and it is not known if LGP2 serves as a primary PRR to detect virus infection or if it serves as a signaling or regulatory cofactor of the other RLRs. Conflicting data have indicated that LGP2 can serve as either a positive or a negative regulator of RLR signaling, depending on the experimental platform or virus infection being assessed [22,23]. Future experimental analyses are needed to address the ligand binding activity and specificity of LGP2 and its role in triggering RLR signaling cascades.

**Figure 1.**
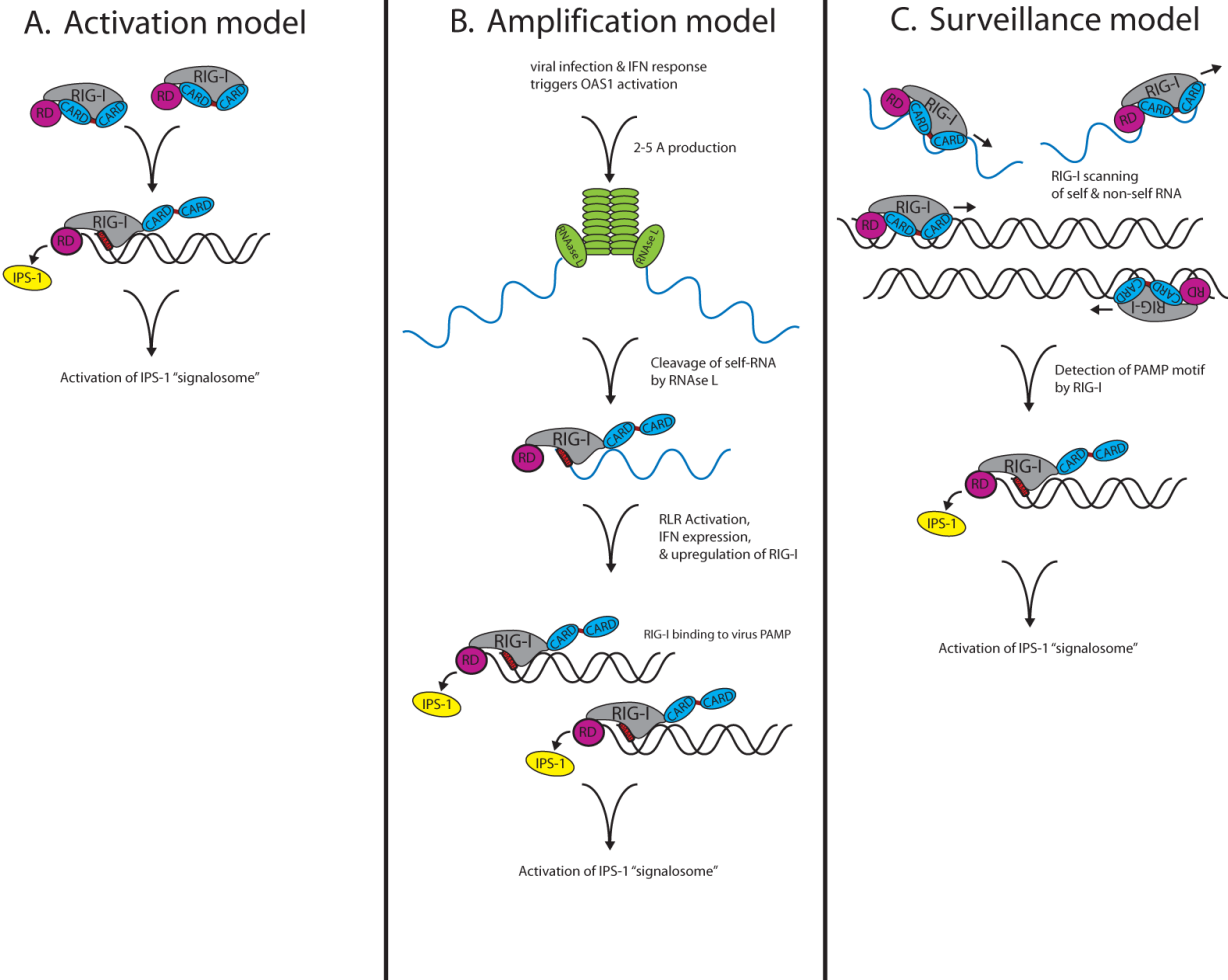
Potential models of RIG-I-like receptors (RLRs) surveillance and activation. (**A**) Activation model. RIG-I is held in a signaling-off conformation through intramolecular interactions between the repressor and CARD domains. During virus infection, RIG-I repressor domain binding to high-affinity RNA ligand PAMP motifs (red box) leads to RIGI activation and the release of the CARD domains for interaction with IPS-1 localized on intracellular membranes, ultimately activating signaling proteins within an IPS-1 “signalosome” that drives the innate immune antiviral response. (**B**) Amplification model. During virus infection, OAS is activated, producing 2–5 A, which in turn, activates RNAse-L cleavage of self and/or viral RNA substrates (delineated in blue), and generating products that then serve as RIG-I and MDA5 ligands. Binding of RIG-I or MDA5 to the RNAse-L-generated RNA products occurs later during virus infection and is supported by increased RLR levels produced as a result of IFN signaling, thus creating an amplification of RLR signaling. (**C**) Surveillance model. Powered by ATP hydrolysis, a single RIG-I molecule or molecular unit repeatedly moves across an RNA molecule, but without dissociating. Once the RIG-I molecule encounters a PAMP motif, it stalls on the RNA, and ATP hydrolysis drives a conformation change to trigger RIG-I signaling activation, thus allowing it to bind to IPS-1 and induce the innate immune response.

Intriguing recent work has shed light on the physical mechanism by which RIG-I conducts its cytoplasmic patrol for viral-derived RNA invaders. The process of how RIG-I patrols the cytoplasm and detects ligand can be described by three different models: Activation, amplification, or surveillance (Figure 1). Each model states that RIG-I is present in the cytoplasm in resting cells, but engagement to a specific PAMP ligand leads to its signaling activation.

- *activation model*. Though RIG-I is present in the cell at a low level, it is not known to signal in the absence of RNA ligand. The activation model is based on the hypothesis that RIG-I is held in a signaling-off conformation through intramolecular interactions of its repressor domain and CARDs. However, during virus infection, RIG-I repressor domain binding to high-affinity RNA ligand, such as 5′ppp/poly-uridine-containing RNA, leads to its release of the CARDs and placement of RIG-I in a signaling-on, activated state. Activated RIG-I is then competent to bind to IPS-1, leading to recruitment and activation of signaling proteins within an IPS-1 “signalosome” that drives the innate immune antiviral response.
- *amplification model*. While the RLRs serve as primary drivers of innate immune signaling, they have also been shown to play an important role in amplifying innate immune signaling through recognition of RNA metabolites that are produced during virus infection by the RNAse-L endoribonuclease. RNAse-L is activated during virus infection through the actions of the 2′,5′-oligoadenylate synthetase (OAS) [24]. Cleavage of self RNA substrates by RNAse-L, such as ribosomal RNA, produces a set of small RNA products that can serve as RIG-I and MDA5 ligands. The binding of RIG-I or MDA5 to these RNA products and the resulting innate immune signaling occurs later during virus infection and is supported by the increased levels of the RLRs that are produced as a result of IFN signaling [25,26]. This late-stage RLR signaling through engagement of RNase-L cleavage products would thereby mediate a critical amplification step of RLR signaling to extend and possibly diversify the innate antiviral state, thus serving to restrict cell-to-cell virus spread.
- *surveillance model*. Recent work by Myong *et al*., 2009, suggests that a single RIG-I molecule or molecular unit can repeatedly move across an RNA molecule without dissociating [27]. This movement is achieved by activation of the DExH box RNA helicase/ATPase domain and ATP hydrolysis to induce translocation of RIG-I along the RNA molecule. Once RIG-I encounters a PAMP motif (such as dsRNA with 5′ppp), RIG-I stalls on the RNA and undergoes the requisite conformation change to trigger signaling activation wherein it is competent to bind to IPS-1 and drive the innate immune response. This model hypothesizes that RIG-I is constantly surveying the cytosol to engage self and non-self RNA molecules alike. By translocating along a given RNA molecule, RIG-I surveys for PAMP motifs that when encountered, lead to RIG-I signaling activation. Thus, RIG-I may be continuously working to identify non-self RNA through this surveillance feature. In addition to triggering RLR-based signaling cascades, this binding and translocation of RIG-I along an RNA molecule could have additional antiviral benefits by inhibiting RNA-protein binding events that are critical to the viral life cycle, thereby directly blocking virus replication. The surveillance model also presents the possibility that RIG-I could aberrantly identify a self-RNA as a ligand. Such an event could aberrantly trigger signaling against self and potentially lead to consequences of autoimmunity or immune toxicity through abnormal innate immune signaling.

## 4. Targeted Therapeutic Applications for RLR Agonists

The functional triggering of innate immune responses, interferon production and signaling, and ultimately interferon-stimulated gene (ISG) induction by RLRs serve to initiate the host immune response to viral infection and provide an important adjuvant action to modulate adaptive immunity through the actions of ISG products. A number of the host factors in antiviral defense and immune response pathways are ISGs that are either not expressed in resting cells or are expressed basally at low levels to provide surveillance against viral infection. These factors are then amplified significantly in response to IFN. For example, in the case of hepatitis C virus (HCV) infection, IFN signaling has been shown to induce an amplification loop in the innate immune response that further increases immune activation [28,29]. In addition to the immediate innate immune response, IFN-α/β signaling also effects longer term immune responses by driving the maturation of dendritic cells and other antigen presenting cells, and supporting the differentiation of specific immune effector cells as well as inducing the production of localized proinflammatory cytokines. These parameters together serve to control cell-mediated defenses and to modulate the adaptive immune response to virus infection [30]. Indeed, studies of gene profiling of liver from HCV-infected chimpanzees have indicated an association between a high level of ISG expression in hepatocytes and the resolution of acute HCV infection [31]. These studies suggest that therapy targeting the activation of innate immune defenses could limit HCV infection to a level that could be controlled by the host immune defenses. While IFN currently serves as the standard of care therapy for HCV infection, the toxic side effects caused by its pleiotropic actions, in part, render it nonspecific and of low overall efficacy. Thus, the development of therapies that trigger specific innate immune programs and lead to select effector gene expression and function could have a revolutionary impact on the control of virus infection and antiviral immune enhancement against both chronic and acute viral infections.

Recent studies have indicated that RLR agonists in particular may have significant therapeutic potential in limiting viral infection. In an *in vitro* model of HCV infection, one study found that triggering RIG-I signaling via HCV PAMP RNA treatment of cells was able to inhibit HCV infection [17]. Similarly, pandemic H1N1 influenza virus infection was limited in an *in vitro* system that activated RIG-I pathways by pre-exposing cells to synthetic 5′-triphosphate RNA [32]. Furthermore, preliminary work using either short dsRNA, short 3′ overhang 5′ppp RNA to trigger RIG-I or long dsRNA to trigger MDA5 has demonstrated that *in vitro* exposure to these ligands induces innate immune programs that can suppress infection by a variety of RNA viruses [11,13,33–35]. Development of RLR-based therapeutics should rapidly advance over the next few years, now that the structure for each RLR ligand binding region has been solved. These structures will enable the design of structure-based small molecule ligands that specifically target and activate the RLRs [35,36]. RLR-targeted therapeutics hold much promise in fighting viral infection as they could induce a specific adjuvant response tailored for enhancing immunity as well as a vaccine response to specific viruses that are subject to RLR recognition.

### 4.1. RLRs Are Intrinsic to NK and DC Function

Several recent studies have focused on understanding the role of RLRs in antigen presenting cell function. Located at the crossroads between the innate and adaptive immune response, dendritic cells (DCs) are a key component of the transition between the immediate and long-term response to viral infection. Since DCs have the unique ability to migrate through the peripherial tissues, blood stream, and lymphoid tissues, their ability to detect virus and alert both innate and adaptive components of the immune system by processing and presenting viral antigens is central to inducing robust host immunity [37]. The role of PRRs in DC biology is being defined, albeit with some conflicting findings. DC maturation and antigen presentation ability is influenced by exposure to IFN-α/β and contributes to directing the differentiation of CD4 T cells toward the Th1 phenotype [38]. Although PRRs are known to act as the second or third signals required by DCs to activate the differentiation of naïve T lymphocytes to effector lymphocytes, the precise PRRs required for this response are not defined and are perhaps cell type and context dependent. Indeed, studies have found that in murine systems, the dependency of DC on RIG-I for inducing antiviral responses varies by the different DC subtypes, with conventional DCs relying on RLR activation and plasmocytoid DCs possibly operating independently of RLRs [15,39]. In contrast, an *in vitro* study using human monocyte-derived DCs found that overexpression of a dominant negative RIG-I construct completely abrogated the induction of cytokines induced by a recombinant Sendai virus vector and only partially inhibited expression of DC maturation markers CD86 and HLA-DR [40]. Recent work has shown that RLRs are differentially expressed in specific DC subsets, wherein CD4+ and CD4−/CD8− DCs, the RLRs may function to program antigen presentation and cytokine production [41]. Thus, future work needs to focus on defining the precise role of RLRs in the various DC populations.

Other studies have indirectly implicated RLR signaling in DC mucosal homing, but these studies have not been able to differentiate between RLR and TLR signaling. Two studies have indicated that either TLR or RIG-I signaling in DCs promotes the mucosal homing of activated lymphocytes [42,43]. Likewise, a zinc-finger ubiquitin-modifying enzyme that inhibits TNF, RIG-I, and TLR signaling pathways, A20, was found to regulate the production of retinoic acid and proinflammatory cytokines by DCs. Moreover, this study found that A20-deficent DCs had an enhanced ability to home to the draining and gut-associated lymphoid tissues after systematic administration in mice [44]. Thus, regulation of innate immune signaling may impose control of immune cell homing through modulation of cytokine production by inflammatory DCs. Obviously, additional work will be needed to assess the relative contribution, if any, of RLRs in the regulation of immune cell and DC homing.

RLRs have also been implicated in enhancing the function of natural killer (NK) cells. NK cells function to kill virus-infected target cells and tumor cells, while also producing proinflammatory cytokines such as TNF and IFN-γ that enhance innate immunity in part by inducing DC maturation. The mature/activated DCs then provide a positive feedback loop with the NK cells by secreting cytokines that enhance NK cell proliferation and cytotoxic potential. RLR receptor signaling has been implicated in enhancing NK/DC cross talk, as optimal IFN-γ production by NK cells requires the cotriggering of RLRs and Toll-like receptor 3 in mDCs and activation of RLRs within NK cells themselves. Indeed, these findings of RLR signaling in NK/DC crosstalk have immediate implications on overall immune responses, as recent studies have found that this cross-talk is critical for support of Th1 differentiation of CD4+ T cells and programming of CTL responses [45–47]. Therefore, RLRs may play an important role in enhancing NK cell function.

### 4.2. RLRs and Inflammatory Bowel Disease

RLRs must maintain the ability to differentiate between self and non-self RNA ligand. As mentioned above, the activation of RLR signaling cascades in response to self-RNA ligand interactions could have severely detrimental effects by inducing an undesired inflammatory reaction, possibly leading to an autoimmune response. In this context, recent work has implicated RLRs in driving the expansion and regulation of regulatory T cells [48]. In light of this finding, RLRs have been implicated as possible mediators of autoimmune diseases and have been associated with inflammatory bowel disease (IBD). IBD is a multifactorial disease characterized by a pathological inflammatory immune response in the intestinal mucosa, including the malfunction of T cells and aberrant cytokine production. Two recent studies have further implicated RIG-I in inflammatory bowel diseases, including Crohn’s disease and ulcerative colitis. One study found that 70% of mice lacking RIG-I gene expression developed a colitis-like phenotype that correlates with inflammatory infiltration and severe damage in the mucosa of the colon [49,50]. The RIG-I-deficient mice had decreases in the size and number of Peyer’s patches, with an increased number of apoptotic B220+ cells within the patches. Moreover, the RIG-I −/− mice had a decreased number of naïve T cells, with an increased number of effector and memory CD4+ and CD8+ T cells in the splenic compartment, a finding which might be related to the suppression of Gαi2 in these mice. The colitis-like phenotype of the RIG-I targeted knockout mouse generated by Wang and colleagues was based on the abrogation of RIG-I expression by disrupting exons 4 to 8 of the *Rig-I* gene [49]. This phenotype directly contrasts with the embryonic lethality of another targeted RIG-I deficient mouse line that was generated by disrupting exons 8 to 10 [39]. The different phenotypes in these mouse lines could be attributed to the potential low expression levels of RIG-I truncation proteins generated by the targeted disruption of the RIG-I gene at different structural locations (the RIG-I −/− mouse displaying the colitis phenotype could possibly express truncated RIG-I with one of the 2 CARD domains, while the embryonic lethal RIG-I −/− knockout mouse would only express a truncated domain containing both CARDs). Despite these different model systems, the colitis phenotype observed in the report by Wang *et al.*, 2007 suggests that RIG-I plays an important role in T cell homeostasis in the gut that is important for maintaining the proper balance between inflammatory activation and tolerogenicity.

In a second study that has substantively showed the involvement of RLRs with IBD, the investigators used an immunohistochemistry approach to assess RIG-I levels in laser-captured microdissected tissues and identified a decrease in RIG-I levels in the intestinal epithelium of Crohn’s Disease patients [51]. Interestingly, this study found no difference in RLR expression in tissue from ulcerative colitis patients. These results were confirmed by RT-PCR and immunoblot analyses. While the pathogenesis of IBD remains to be understood, the mouse and human-based studies have indicated that abrogation or disregulation of RLR signaling may play a large part in the etiology of IBD. Future studies will need to address the specific contribution of RLRs to IBD pathogenesis in order to determine if RLR-targeted therapeutics would have clinical application for IBD patients.

### 4.3. RLRs and Immunotherapy

Therapies that trigger the innate immune response could enhance the communication between the innate and adaptive immune programs, and therefore could have potential as immune adjuvants that enhance immune response function and long term immunity against viruses and other disease-causing agents. In this light, agonists that trigger RLRs or downstream signaling nodes have the potential to be developed as the next generation of vaccine adjuvants. Indeed, the N-CARD domain of the downstream RLR activator, IPS-1, has demonstrated adjuvant activity by improving humoral and cellular responses to protein vaccines [52]. Another study found that the establishment of CD8 T cell memory after vaccination with a poly(I:C) adjuvant requires MDA5 activation [53]. Finally, the most recent study has directly assessed the ability of a RIG-I agonist to function as an adjuvant by generating an H5N1 influenza virus DNA vaccine vector that co-expressed a RIG-I agonist. Mice given intramuscular injections with these naked DNA vaccines demonstrated increased HA-specific serum antibody binding avidity [54]. However, the effects of this vector system on cellular immunity were limited, with CD8 cell epitopes only producing a less than 2-fold induction of IL-2, which is the cytokine responsible for increasing the number of mature CD8 T cells and enhancing CD8 immune cell memory. Additional work is needed to determine if the high dose of immunogen or tissue specific effects could explain the lack of significant induction of CD8 and CD4 cellular immune response to HIVgag in this system. Regardless, these studies demonstrating the ability of a RIG-I agonist containing influenza vaccine to stimulate IL-2 and improve binding HA-antibody avidity indicates that RLRs are indeed promising targets for adjuvant developments.

On a cautionary note, any unbridled activation inflammatory pathways that go unchecked can lead to cytokine storms that are fatal to the host. Thus, perturbations of the RLR pathways that go unmediated could have severe implications in maintaining the ability to mediate a healthy immune response. In this light, any therapeutic intervention in the RLR pathway must be carefully monitored to ensure that the responses induced can be adequately controlled.

Moreover, many viruses have mechanisms to evade the innate immune response, particularly the RIG-I pathway. These viral evasion mechanisms have been described in detail elsewhere [55–59], but generally block RLR signaling by preventing RLR recognition of viral RNA or disrupting the downstream signaling components. It is important to note that the evasion mechanisms used by viruses can allow infection to proceed despite triggering an overall antiviral immune response. Thus, RLR-based therapeutic design must take into account these specific viral evasion strategies in order to provide the greatest clinical benefit.

In summary, RLR function is essential for host control of virus infection by activating antiviral mechanisms of innate immunity and enhancing the adaptive immune response. Harnessing the capacity of RLRs to initiate powerful antiviral responses by RLR-targeted therapies has the potential to radically transform antiviral approaches as well as potentially serve as the basis for the next generation of vaccine adjuvants. Future studies that focus on understanding the role of RLRs in DC biology as well as proinflammatory diseases and cancer will enhance our ability to fully utilize the therapeutic potential of RLR agonists.

## Figures and Tables

**Table 1.** RIG-I-like receptors (RLRs) Ligands.

	**RIG-I**	**MDA5**
**Viral Nucleic Acid Structure**	5′-ppp RNA + poly-uridine RNAase-L products	Long ds RNA RNA replication intermediates RNAse-L products
**Synthetic Ligands**	*In vitro* transcribed RNA, see [60]	poly(I:C)
**Virus**	Reovirus Dengue virus West Nile virus Rotavirus Sendai virus Vesicular stomatitis virus Respiratory syncytial virus Influenza A virus Ebola virus Hepatitis C virus Japanese encephalitis virus Newcastle disease virus RNA pol III transcription products	Reovirus Dengue virus West Nile virus Rotavirus Sendai virus Polio virus Encephalomyocarditis Mengo virus Theiler’s virus murine norovirus
